# Development and validation of deep learning based embryo selection across multiple days of transfer

**DOI:** 10.1038/s41598-023-31136-3

**Published:** 2023-03-14

**Authors:** Jacob Theilgaard Lassen, Mikkel Fly Kragh, Jens Rimestad, Martin Nygård Johansen, Jørgen Berntsen

**Affiliations:** Vitrolife A/S, Aarhus, Denmark

**Keywords:** Biomedical engineering, Machine learning, Computational biology and bioinformatics

## Abstract

This work describes the development and validation of a fully automated deep learning model, iDAScore v2.0, for the evaluation of human embryos incubated for 2, 3, and 5 or more days. We trained and evaluated the model on an extensive and diverse dataset including 181,428 embryos from 22 IVF clinics across the world. To discriminate the transferred embryos with known outcome, we show areas under the receiver operating curve ranging from 0.621 to 0.707 depending on the day of transfer. Predictive performance increased over time and showed a strong correlation with morphokinetic parameters. The model’s performance is equivalent to the KIDScore D3 model on day 3 embryos while it significantly surpasses the performance of KIDScore D5 v3 on day 5+ embryos. This model provides an analysis of time-lapse sequences without the need for user input, and provides a reliable method for ranking embryos for their likelihood of implantation, at both cleavage and blastocyst stages. This greatly improves embryo grading consistency and saves time compared to traditional embryo evaluation methods.

## Introduction

Prioritizing embryos for transfer and cryopreservation is a long-standing challenge in the field of in vitro fertilization (IVF) with both academic and commercial research dedicated to its resolution. When multiple good quality embryos are available, selection of the embryo with the highest likelihood of implantation will shorten the time to pregnancy and ultimately live birth. Traditionally, embryo evaluation has been carried out by manual inspection of either static microscope images or time-lapse videos of developing embryos. Scoring systems based on morphological and morphokinetic annotations have been used to rank embryos within patient cohorts as well as to decide, which embryos to discard and which to transfer and/or cryopreserve. In recent years, however, the use of artificial intelligence (AI) to evaluate embryos has shown promise in both automating the assessment and potentially surpassing the ranking performance of manual inspection^1^.

Increasingly, blastocyst transfer has become the preferred development stage for transfer^2^, and most AI models for embryo evaluation specifically address embryos cultured to day 5 or later^3–6^. However, blastocyst culture generally results in a lower number of embryos to choose from, and for patients with poor embryo development, cleavage-stage transfers may be preferred if there is a risk of a cancelled cycle^2^. Few studies have focused on both cleavage-stage and blastocyst transfers^7,8^. Erlich et al.^7^ propose a combined model for handling day 3 and day 5 transfers, by predicting a score for each image in a time-lapse sequence. Scores from previous images in the sequence are then aggregated temporally. The authors claim that the method provides continuous scoring regardless of development stage and time, and that it outperforms the manual morphokinetic model, KIDScore D3^9^. However, as they only evaluate on day 5 transfers, they ignore the possibility that embryo characteristics may differ in their importance on day 3 and day 5 transfers. Kan-Tor et al.^8^ also propose a combined model for handling day 3 and day 5 transfers, by first predicting scores for non-overlapping temporal windows, and then aggregating scores from previous windows in the sequence using logistic regression. The authors show both discrimination and calibration results together with subgroup analyses on patient age and clinics for day 5 transfers. However, for day 3 transfers, only the overall discrimination performance is presented. Therefore, the calibration and generalization performance on day 3 embryos across subgroups such as patient age and clinics remains to be seen.

**Figure 1:**
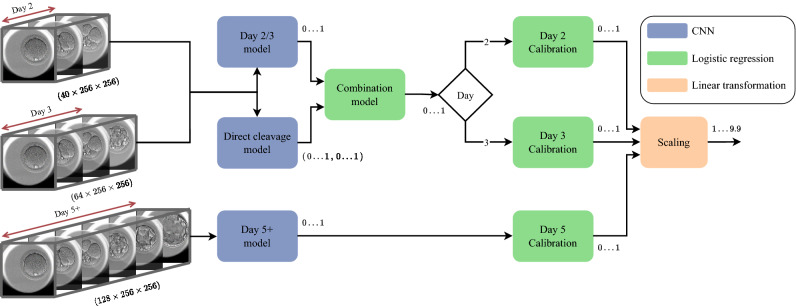
iDAScore v2.0. Two separate tracks handle day 2/3 and day 5+ embryos. The first track consists of two 3D convolutional neural networks (CNN) that predict implantation potential and direct cleavages, followed by separate calibration models for day 2 and 3. The second track consists of a 3D CNN that predicts implantation potential followed by a day 5+ calibration model. Finally, scores from both tracks are scaled linearly to the range 1.0–9.9.

In addition to day of transfer, current AI models often deviate in how they approach automation. Some methods assume manual preselection by embryologists and can thus be categorized as semi-automated. These are methods that have only been trained on transferred embryos and therefore generally have not seen embryos of poor quality^4–6^. Other methods approach full automation by training on all embryos, regardless of whether they were transferred or not. These methods rely on other labels than pregnancy for the non-transferred embryos such as manual deselection by embryologists (discards), results of preimplantation genetic testing for aneuploidy (PGT-A), or morphokinetic and/or morphological annotations^3,7,10^. For an AI model to be both fully automated and superior in ranking performance on previously transferred embryos, both aspects need to be evaluated^1,11^. The performance of both transferred embryos with known implantation data (KID) and non-transferred embryos of different qualities and development stages needs to be evaluated in order to ensure general prospective use. In this study, we describe the development and validation of a fully automated AI model, iDAScore v2.0, for embryo evaluation on day 2, day 3 and day 5+ embryos. As in our previous work^3^, the model is based on 3D convolutions that simultaneously identify both spatial (morphological) and temporal (morphokinetic) patterns in time-lapse image sequences. However, whereas our previous work only dealt with ranking performance, in this study, we also calibrate the model to obtain a linear relationship between model predictions and implantation rates. We train and evaluate our model on an extensive and diverse dataset including 181,428 embryos from 22 IVF clinics across the world. On independent test data, we present both discrimination and calibration performance for embryos transferred after 2, 3 and 5+ days of incubation, individually, and compare with iDAScore v1^3,12,13^ and the manual morphokinetic models, KIDScore D3^9^ and KIDScore D5 v3^14^. We also present discrimination results for a range of subgroups including patient age, insemination method, transfer protocol, year of treatment, and fertility clinic. Finally, we perform temporal analyses on score developments from day 2 to 5 to illustrate improvements over time in discrimination performance, temporal changes in ranking, and relation to common morphokinetic parameters used for traditional embryo selection (Fig. 1). To the best of our knowledge, our work presents the first AI-based model for ranking embryos from day 2 to day 5+, and is the first study to present calibration curves and subgroup analyses on transferred cleavage-stage embryos.

## Methods

The data used in this study are retrospective and anonymized. In Denmark, the study described was deemed exempt from notification to the National Committee on Health Research Ethics according to the Act on Research Ethics Review of Health Research Projects (Consolidation Act No. 1338 of September 1, 2020). Informed consent was obtained from all participants. All methods were conducted in accordance with the relevant guidelines and regulations.

### Study design

**Figure 2:**
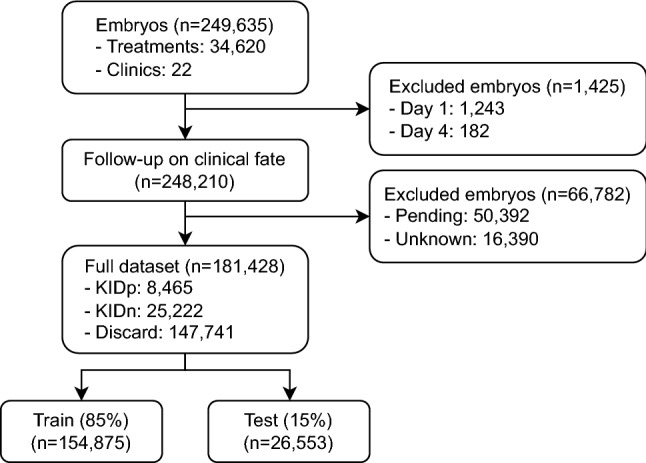
Flowchart of the study design.

The study was a multi-centre retrospective cohort study consisting of 249,635 embryos from 34,620 IVF treatments carried out across 22 clinics from 2011 to 2020 with both single embryo transfers and multi embryo transfers. As the study focused on day 2, day 3 and day 5+ transfers, day 1 ($\text {n}=1243$) and day 4 ($\text {n}=182$) embryos were excluded, corresponding to embryos incubated less than 36 hours post insemination (hpi) and embryos incubated between 84 hpi and 108 hpi. Furthermore, embryos without known clinical fate were excluded, as their clinical outcomes were either unknown ($\text {n}=16,390$) due to follow-up loss ($\text {n}=3192$), a multi embryo transfer where some, but not all, embryos resulted in positive outcomes ($\text {n}=13,198$), or because they were still cryopreserved at the time of data collection and thus had pending outcomes ($\text {n}=50,392$). After data exclusion, 181,428 embryos remained, of which 33,687 were transferred embryos with known implantation data (KID) measured by the presence of a fetal heartbeat, and 147,741 were discarded by embryologists either due to arrested development, failed fertilization, aneuploidy, or other clinical deselection criteria. Finally, the dataset was split into training (85%) and testing (15%) on treatment level, ensuring that all embryos within a given treatment were either allocated to training or testing. While this split-strategy allows cohort-analyses on the test set, it also mitigates certain types of bias, as the AI model cannot benefit from overfitting to individual patients in the training set. A flowchart illustrating patients, exclusion of data points (embryos), and division into training and test subsets is shown in Fig. 2.

Table 1 shows the specific number of discarded embryos and KID embryos with positive (KID+) and negative (KID-) outcomes for each day in the training and test sets. Supplementary Table S3, Supplementary Table S4 and Supplementary Table S5 contain further details on patient age, clinical procedures, and embryos for each clinic in the data subsets of day 2, day 3, and day 5+ embryos, respectively.

**Table 1:** Datasets for training and testing the model.

(a) Training data				
	Day 2	Day 3	Day 5+	Total
Discarded	12,627	14,121	99,288	126,036
KID-	7095	4876	9656	21,627
KID+	1453	1075	4684	7212
Total	21,175	20,072	113,628	154,875

(b) Test data				
	Day 2	Day 3	Day 5+	Total
Discarded	2029	2491	17,185	21,705
KID-	1165	809	1621	3595
KID+	258	194	801	1253
Total	3452	3494	19,607	26,553

### Image data

All embryos were cultured in EmbryoScope$^{\textrm{TM}}$, EmbryoScope$^{\textrm{TM}}$+, or EmbryoScope$^{\textrm{TM}}$ Flex incubators (Vitrolife A/S, Aarhus, Denmark). The incubators acquired time-lapse images during embryo development according to specific settings in each clinic. For EmbryoScope$^{\textrm{TM}}$ incubators, microscope images of 3–9 focal planes at a pixel size of 500$\times$500 were acquired every 10–30 min. For EmbryoScope$^{\textrm{TM}}$+ or EmbryoScope$^{\textrm{TM}}$ Flex incubators, microscope images of 11 focal planes at 800$\times$800 pixels were acquired every 10 minutes.

### Model development

To predict embryo imp lantation on day 2, 3 and 5+, we developed a combined AI model consisting of several components. Figure 1 shows a flowchart of the model. If an embryo is incubated more than 84 hpi, raw time-lapse images from 20–148 hpi are fed to a 3D convolutional neural network (CNN) that outputs a scalar between 0 and 1 (Day 5+ model). This CNN is identical to the one in our previous work [3], however with a few key differences to the training methodology. A full explanation of the differences is found in Supplementary Methods A. If, however, the embryo is incubated less than 84 hpi, images from 20 to 84 hpi are fed to two separate CNN models that evaluate respectively the overall implantation potential (Day 2/3 model) and the presence of direct cleavages from one to three cells and from two to five cells (Direct cleavage model). The day 2/3 model outputs a scalar between 0–1, and the direct cleavage model outputs two scalars (one for each type of direct cleavage) between 0 and 1. A logistic regression model then combines the three outputs into a single scalar. Finally, outputs from either day 2/3 or day 5+ are calibrated individually for each day on KID embryos to obtain a linear relationship between scores and implantation rates and to remove any calibration bias introduced by our training methodology. At this point, the scores are estimates of pregnancy probabilities representative of the average patient population (including various diagnostic profiles), as opposed to individualized probabilities for each patient. Therefore, to avoid confusing probabilities as being individualized, the calibrated scores are ultimately rescaled to the range 1.0–9.9, similar to the range used in our previous work^3^ and by the manual morphokinetic model, KIDScore D5v3^14^. During training, discarded embryos are treated as KID- but sampled such that KID- embryos and discarded embryos are equally represented. Using discarded embryos during training does not negatively influence the capability to discriminate between KID+ and KID- but improves the model’s capability to categorize embryos into usable or discarded embryos^15,16^.

For more details on model architectures, training methodology including data sampling, preprocessing and augmentation strategies as well as individual results for the components, see Supplementary Methods A.

### Model validation

Internal validation was used to evaluate the predictive performance of the model on test data in terms of discrimination and calibration^1,17^. We denote evaluations on KID embryos as KID+ vs KID- and evaluations on all embryos as KID+ vs KID- plus discarded. Discarded embryos are therefore assumed to be KID- in evaluations on all embryos. The area under the receiver operating characteristic curve (AUC) was used to quantify discrimination and reported with 95% confidence intervals using DeLong’s algorithm^18^. Tests for significant differences in AUC were performed using either paired or unpaired two-tailed DeLong’s test^18^. Bonferroni-adjusted p-values were used for reporting significant differences of multiple comparisons. Calibration was assessed graphically using observed implantation rates in grouped observations of similar predictions (quantiles) and Loess smoothing^19^.

## Results

We present the combined discriminatory performance in terms of AUC for iDAScore v2.0 in (a) along with intermediate results for each component from Fig. 1. For each day (2, 3 and 5+), the table lists the AUC for all embryos (KID+ vs. KID- plus discarded) and for KID embryos (KID+ vs. KID-). The AUCs on day 2, 3 and 5+ were 0.861, 0.872 and 0.954 for all embryos and 0.669, 0.621 and 0.707 for KID embryos. (b) provides a comparison with two manual scoring systems, KIDScore D3^9^ and KIDScore D5 v3^14^, as well as our previous work, iDAScore v1^3^, on embryos in the test set that had manual morphological and morphokinetic annotations required by KIDScore. iDAScore v1 was evaluated on the test set used for iDAScore v2.0. As this includes training samples from v1, the iDAScore v1 performance may be overestimated. On day 3, the AUCs of iDAScore v2.0 and KIDScore D3 on KID embryos were 0.608 and 0.610, with no significant differences according to a paired DeLong’s test ($p=0.92$). On day 5+, however, the AUCs of iDAScore v2.0 and KIDScore D5 v3 on KID embryos were 0.694 and 0.645 and significantly different ($p<0.001$). When comparing iDAScore v2.0 against iDAScore v1 on KID embryos, AUCs were 0.694 and 0.672 and also significantly different ($p=0.045$).

**Figure 3:**
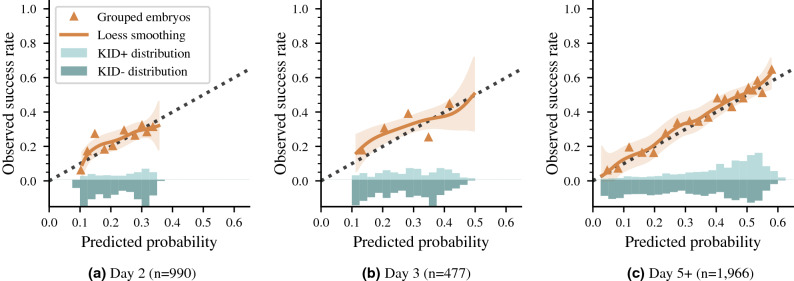
Calibration curves linking predicted probabilities to actual success rates for day 2, 3 and 5+ single embryo transfers, respectively. The dotted line represents perfect calibration. Grouped observations (triangles) represent success rates for embryos grouped by similar predictions. Loess calibration (solid line) represents a smoothed estimate of observed success rates in relation to model predictions. The shaded area is the 95% confidence interval. The relative distributions of scores for positive and negative pregnancy outcomes are shown at the bottom of the graph.

The calibration performance of iDAScore v2.0 on single embryo transfers is shown in Fig. 3 for day 2, 3 and 5+, individually. In general, there is a good agreement between predicted probabilities and observed implantation rates. Comparing the three curves, we see that both the ranges of predictions and success rates increased from day 2 to 3 and from day 3 to 5+. That is, the best day 3 embryos had higher scores and a higher implantation rate than the best day 2 embryos. And on day 5+, we observe both the highest and lowest scores as well as the highest and lowest implantation rates. This suggests that with more information available on the blastocyst stage, the model can more confidently assign a probability of implantation, ranging from around 6% for the lowest scores up to 65% for the highest scores on day 5+. As these predictions were made based on time-lapse images alone, however, they represent average patient probabilities and not individualized patient probabilities. To predict probabilities at patient-level, additional characteristics such as patient demographics and clinical practice should be included in the calibration procedure and analysis^1,20^. However, these aspects are outside the scope of this work (Table 2).

**Table 2:** AUCs on the test set for the different model components across days of incubation.

	(a) Full test set					
	Day 2		Day 3		Day 5+	
	All ($\text {n}=3452$)	KID ($\text {n}=1423$)	All ($\text {n}=3494$)	KID ($\text {n}=1003$)	All ($\text {n}=19,607$)	KID ($\text {n}=2422$)
Day 2/3	.856 [.840–.872]	.663 [.630–.697]	.862 [.844–.879]	.611 [.569-.654]	–	–
Combination	.861 [.844–.877]	.669 [.635–.703]	.872 [.854–.890]	.621 [.579–.662]	–	–
Day 5+	–	–	–	–	.954 [.950-.958]	.707 [.686-.728]
iDAScore v2.0	.861 [.844–.877]	.669 [.635–.703]	.872 [.854–.890]	.621 [.579–.662]	.954 [.950–.958]	.707 [.686-.728]

	(b) Comparisons on subset of test set with annotations required by KIDScore D3 and D5					
	Day 3	Day 5+				
	KID (n = 800)	KID (n = 1175)				
KIDScore D3	.610 [.569–.651]	–				
KIDScore D5	–	.645 [.613–.676]				
iDAScore v1	–	.672 [.641-.703]				
iDAScore v2.0	.608 [.562–.654]	.694 [.664–.724]				

*All* denotes KID+ vs. KID- plus discarded embryos, whereas *KID* denotes KID+ vs. KID- embryos. All AUCs are reported with 95% confidence intervals in brackets. (a) lists results on the full test set from Table 1b, whereas (b) compares performance with KIDScore D3, KIDScore D5 and iDAScore v1 models on embryos that have manual annotations available as required by KIDScore.

### Subgroup analysis

We investigate the generalization performance of iDAScore v2.0 across different patient demographics and clinical practices by performing subgroup analysis on KID embryos for the following parameters: patient age (<30, 30–34, 35–39 and >39 years), insemination method (IVF and ICSI), transfer protocol (fresh and cryopreserved), treatment year (<2015, 2015–2016, 2017–2018, >2018), and clinics (1–22). The results are available in Supplementary Table S6 that lists the number of KID embryos and corresponding AUCs for each subgroup. We found that on day 5+, AUCs for the age group > 39 were significantly higher than for all other age groups ($p<0.03$) using unpaired DeLong’s test. For the transfer protocol, we found a significant difference between AUCs for fresh and cryopreserved transfers on day 5+ ($p=0.03$). We found a significant difference between treatment years > 2018 and 2015–2016 on day 2 ($p=0.02$). While this difference in theory could indicate temporal biases due to improvements in IVF treatments over time, it may also represent differences between clinics, as not all clinics contributed data across all years. Differences between individual clinic AUCs were significant in multiple cases. On day 5+, this includes clinic 18 vs 20 ($p=0.02$) and clinic 21 vs 1, 5, 10, 11, 16, 20 ($p<0.05$). Clinic 21 thus performed significantly different to most other clinics on day 5+ and had the lowest AUC of 0.516, indicating close to random discrimination performance. This may be due to a variety of factors. Most importantly, clinic 21 was the only clinic to perform PGT-A routinely, and thus only transferred euploid embryos. It is expected that this would lower the AUC of any selection algorithm that correlates with euploidy. When evaluating the performance for discriminating between euploid ($n=178$) and aneuploid ($n=269$) embryos from clinic 21, a considerably higher AUC of 0.68 for iDAScore v2.0 was evident, in line with expectation.

### Predictive performance over time

**Figure 4:**
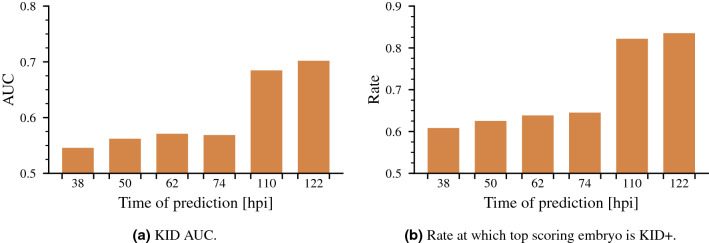
Evaluation of predictive performance over time.

We assessed the predictive performance over time by evaluating the model at 12 hour intervals from 38–122 hpi. The performance was not assessed between 84–108 hpi as the training data does not include day 4 transfers. The model was evaluated on the day 5+ test set as this is the only set that allows evaluation at all points in time. Two different evaluation methods were used: The AUC for predictions on KID embryos at different times on the day 5+ test set in Fig. 4a, and the rate at which the highest scoring embryo in each treatment is KID+ in Fig. 4b. For the second method, treatments without any KID+ were excluded and discarded embryos were included in all treatments.

There is a significant improvement in performance when going from predictions on cleavage stage transfers to predictions on blastocyst stage transfers. This is in agreement with previous reports^7,8^. There is a small improvement in predictive performance for later predictions on day 2 and day 5, while day 3 appears to have the same performance.

### Correlation with manually annotated morphokinetics

We evaluated the biological explainability of iDAScore v2.0 by estimating the average implantation rate for groups of embryos with similar morphokinetic parameters and comparing them with the average implantation rate predicted by iDAScore v2.0. The morphokinetic parameters are $t_{\textrm{PNf}}$, $t_2$, $t_4$, $t_8$, $t_3-t_{\textrm{PNf}}$, and $t_5-t_3$. The morphokinetic parameters were manually annotated by trained embryologists working at their respective clinics. We estimated the average implantation rate by grouping embryos with a morphokinetic parameter in five- or ten-hour intervals. For example, for estimating the implantation rate of embryos with a $t_2$ of 30 hpi, we compute the mean implantation for embryos with a $t_2$ between 27.5 hpi and 32.5 hpi and compute its 95% confidence interval along with the mean iDAScore v2.0 prediction after 44, 68, and 116 hpi. Only embryos with an annotation of each parameter were included in the analysis. Many clinics select embryos for transfer based on morphology and morphokinetics which results in a limited amount of data with known implantation outside the range of normal development. Therefore, we included discarded embryos in this analysis under the assumption that none of the discarded embryos would have implanted.

**Figure 5:**
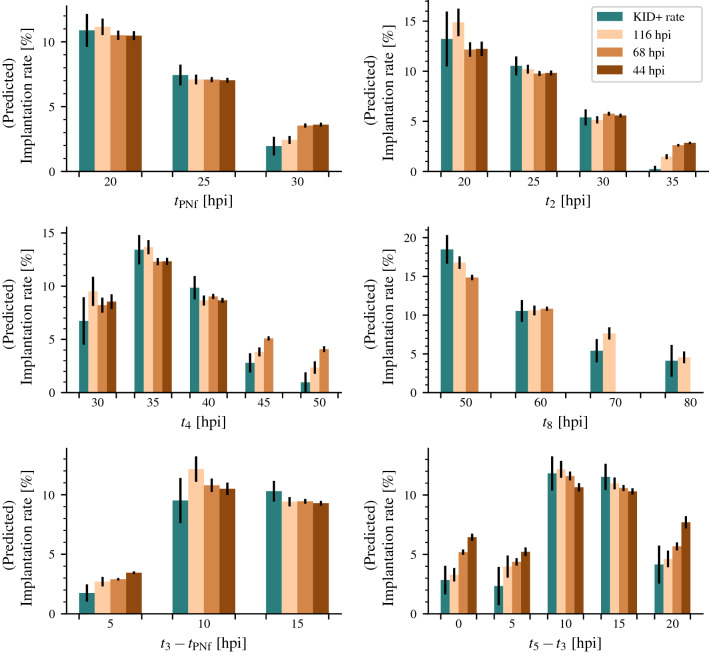
Evaluation of biological explainability of iDAScore v2.0 that shows a comparison between the estimated implantation likelihood (KID+ rate) for embryos with similar morphokinetic parameters and their predicted implantation rate at 116 hpi, 68 hpi, and 44 hpi. The evaluation uses the day 5+ test set with an assumption that deselected embryos have an implantation likelihood of 0%. The bars denote the 95% confidence interval. There is a good concordance between the predicted implantation rate and the actual implantation rate with slight overestimation at the extremes.

There is a bias in which clinics annotated the above morphokinetic parameters, therefore the model output was re-calibrated using only data with annotations to isolate the response to the morphokinetic parameters. The comparison between the implantation rate for embryos with similar morphokinetic parameters and the prediction is shown in Fig. 5.

The predicted implantation rate is often within the confidence interval of the actual implantation rate for different timings of the morphokinetic events. The 116 hpi predictions are closest to the actual implantation rate compared to the predictions at 68 hpi and 44 hpi. Some predictions at 44 hpi for embryos with a $t_5-t_3$ of 20 have a $t_5$ that is later than 44 hpi and thus the model has no chance of even seeing $t_5$. For the other morphokinetic parameters there is a slight overestimation of the implantation rate at the extremes, which is more pronounced for predictions at 44 hpi and 68 hpi than at 116 hpi. Overall, the changes in the predicted implantation rates follow the trend for the actual implantation rates. This suggests the model has either learned to recognise the morphokinetic parameters or some features that heavily correlate with the morphokinetic parameters.

## Discussion

Embryo selection is the task of prioritizing the order of transfer and which to cryopreserve or discard among all available embryos from a patient. When automating and potentially improving this task using AI, it is important to be aware of potential biases introduced. If training and evaluation are carried out solely on transferred embryos, the dataset will be biased towards good quality embryos, and the model may not generalize to embryos of poor quality. In practice, this means that a manual preselection by embryologists of the embryos that are good enough to be transferred is implicitly assumed. To avoid this issue, we included discarded embryos in the training set and balanced their contribution by oversampling the transferred embryos, just as in our previous work^3^. Berntsen et al.^15^ and Erlich et al.^16^ show that using discarded embryos during training does not negatively influence the capability to discriminate between KID+ and KID- but improves the models capability to categorize embryos into usable or discarded embryos. Similar approaches have been proposed by others by including non-transferred embryos during training using pseudo soft labeling^7^, or by adding aneuploid embryos determined with PGT-A testing to the negative class^10^.

Another source of bias can occur when assuming the selection criteria are independent of the day of transfer. That is, cleavage-stage embryo characteristics may not have the same importance or interactions affecting prediction outcomes for day 2 and day 5 transfers. Therefore, training or evaluating cleavage-stage models on outcomes from blastocyst transfers as described by Erlich et al.^7^ may bias the results. In our preliminary experiments, we observed that separate AI models for cleavage-stage embryos and blastocysts resulted in a higher performance than when using both a single combined model and individual models for each day. This suggests that the optimal cleavage-stage characteristics for selecting an embryo to transfer on day 2 or 3 may not actually be the same as for selecting (2–3 days ahead of time) which embryo to transfer on day 5. Therefore, it is essential to evaluate AI model predictions based on actual transfer day as was presented in our validation. As an exception, we carried out temporal score analyses on day 5+ embryos, as these were the only embryos that could be evaluated across the entire development period from 20–148 hpi. Here, day 5+ outcomes were assumed also to be representative of cleavage-stage outcomes, which is a limitation of the analyses.

In a comparison with the two manual scoring systems, KIDScore D3^9^ and KIDScore D5 v3^14^, as well as our previous work on iDAScore v1^3^, we found no significant performance difference between iDAScore v2.0 and KIDScore D3 while iDAScore v2.0 significantly outperformed both KIDScore D5 v3 and iDAScore v1. As such, iDAScore v2.0 seems to perform as well as KIDScore D3 on selecting embryos for transfer on day 3, but without requiring any manual annotations. The results on day 5+ suggests that the increased amount of training data and slightly modified training strategies from v1 to v2.0 have improved the model performance significantly.

We addressed the performance across various potential confounders by presenting subgroup comparisons of AUCs for the variables age, insemination method, transfer protocol, year of treatment, and IVF clinic. Here, we found significant differences between the age group > 39 and all other age groups. We observed a general trend of higher AUCs with increased age. This observation aligns well with other reports that have shown AUCs to increase with age^6,7,21^. This is expected, since age is a strong independent predictor of pregnancy. However, including age as an input to the AI model is inadvisable, since increases in AUCs do not necessarily reflect improvements in ranking performance within single patient cohorts^1,22^. Erlich et al.^7^ speculated that differences in endometrial receptivity with age may influence label noise and thus subgroup AUCs. The trend may also be caused by general differences in embryo qualities between younger and elder women. Younger women typically have transferred embryos of high quality, whereas elder women more often have transfers with poor or medium quality embryos. The wider distribution of embryo qualities for elder women thus results in higher AUCs. To eliminate such biases, we also evaluated embryo ranking for different age groups at treatment level. For this, we calculated the rate at which the highest scoring embryo in each treatment was a KID+. For the same age groups <30, 30–34, 35–39 and >39 years, the rates were 0.861, 0.836, 0.849, and 0.827, showing a slight decrease in performance with age, if anything. This suggests that the higher AUCs with increased age are not caused by a lack of model generalization but possibly by a bias in the distribution of embryo quality for transferred embryos for elder women.

Our subgroup comparisons also revealed significant differences between transfer protocols, certain years of treatment, and certain clinics. While this may indicate generalization weaknesses, it may also originate from other biases in the dataset, such as different age distributions across clinics and years. It may be relevant to adjust for known confounders such as age when comparing other subgroups. Theoretically, this should help isolate variables and provide less biased subgroup performance evaluations.

There were increases in performance for both AUC and the rate of KID+ scoring highest in each treatment from later culture day predictions. The most significant performance increase came when going from predictions at the cleavage stage to the blastocyst stage, while the intraday performance was only slightly higher for day 2 and day 5+ and the same for day 3.

We compared the estimated implantation rate and the predicted implantation rate for embryos grouped by similarity for various morphokinetic parameters to address the biological explainability of the model. Here we assume that none of the discarded embryos would implant which is not guaranteed to be correct. As we calibrate using the same assumptions, it is unlikely to bias the comparison. It does, however, result in implantation rates that are significantly lower than those of transferred embryos, making the actual values uninteresting. In general, we see a good concordance between the estimated and the predicted implantation rates, except for very late $t_2$ and $t_4$ predictions along with predictions after 44 hpi and 68 hpi for embryos with a $t_5-t_3$ of 0 hpi and 5 hpi. As this is not an issue for $t_3-t_{\textrm{PNf}}$, it is likely a result of the performance difference between predicting direct cleavages from one to three cells and direct cleavages from two to five cells, shown in Supplementary Table S2. For embryos with a late $t_2$ and $t_4$, the implantation rate is overestimated but still gives lower implantation likelihood than for embryos with earlier $t_2$ and $t_4$, which suggests that it does not impact the ability to rank embryos.

A limitation of the present study is that it used internal validation to evaluate generalization performance, both overall and across subgroups. To evaluate actual generalization performance of new clinics that have not taken part in the training process, external validation should be performed. In order to eliminate potential biases caused by retrospective evaluation, a prospective study should be used to reveal the actual performance in a clinical setting. Currently, an ongoing randomized controlled trial (The VISA Study, NCT04969822) is investigating how iDAScore v1^3^ performs compared to manual grading on day 5 embryos.

## Supplementary Information

Supplementary Information 1.

Supplementary Information 2.

## Data Availability

The data that supports the findings of this study are available as supplementary material.
