# Supplementary material for: Development and validation of deep learning based embryo selection across multiple days of transfer
Source: Sci Rep. 2023 Mar 14;13:4235. doi: 10.1038/s41598-023-31136-3 (PMC10015019; doi:10.1038/s41598-023-31136-3)
Supplement: Supplementary file 1 — Supplementary Information 1. [file 41598_2023_31136_MOESM1_ESM.pdf]

Supplementary Methods

A iDAScore v2.0 model description

The iDAScore v2.0 model is composed of multiple components as shown in Figure 1. In this section, each of the components are described in detail including architectures, training methodology and individual results.

A.1 Day 5+

The day5+ model takes as input 128 center-focal images at 1 frame/hour from 20-148 hpi. All images are resized to a resolution of 256×256 pixels. If an embryo is cultured less than 148 hpi, blank frames are appended so the sequences are always 128 frames long. The model is based on the inflated 3D convolutional network (I3D) [23] and has the same architecture and data augmentation as in our previous work [3] with the following exceptions to the data augmentation strategies:

- Random truncation up to two frames from the end of the video instead of random truncation between 108 hpi and 140 hpi.
- Random contrast between 0.85 and 1.15.
- No use of random rotation.

These new strategies as well as all other hyper-parameters were found using 5-fold cross-validation on the training set in Table 1a.

The training process is divided into two stages using knowledge distillation [24]. First, we split the training set into five folds in a similar manner to 5-fold cross-validation and train a teacher model on each fold. Each teacher model is trained using the Adam optimizer [25] with β_1 and β_2 set to 0.9 and 0.999, respectively, with a one-cycle learning rate schedule [26] and an initial learning rate of 5e-5 and a maximum learning rate of 5e-4. The training data are sampled such that KID+, KID- and discarded day 5+ embryos represent 50%, 25%, and 25% of a batch, respectively. We use a batch size of 32 and focal loss [27] with an α of 0.5 and a γ of 0.5. In the loss function, KID+ are represented by values of 1, and KID- and discarded embryos are represented by values of 0. Each model is trained with 34,080 batches.

Each teacher then predicts on all its training samples (4/5 of the full training set). With five folds, this results in four predictions for each embryo. A single student model is trained to mimic the average of these predictions through a modification of the teacher's loss function:

$$L = 0.9H(y,\bar{y}) + 0.1H(y,d) \quad (1)$$

where H is the cross-entropy, y is the student model prediction, $\bar{y}$ is the average prediction of the teacher models, and d is the outcome label. The student model is trained using the same hyper-parameters as the teacher models, except for an initial learning rate of 1e-4 and a maximum learning rate of 1e-3. The student model is trained with 42,624 batches.

A.2 Day 2/3

Based on 5-fold cross-validation on the training set in Table 1a, it was found that separate models for day 2/3 and day 5+ embryos resulted in higher performance than both a single combined model and individual models for each day.

Day 2/3 model

The day 2/3 model is similar to the day 5+ model, except that it takes as input 64 center-focal images at 1 frame/hour from 20-84 hpi. And if an embryo is cultured less than 84 hpi, blank frames are appended so the sequences are always 64 frames long.

The day 2/3 model is trained using the same two-stage process of knowledge distillation [24] as the day 5+ model. Each teacher model is trained using the same hyper-parameters as the day 5+ model, except for an initial learning rate of 3e-4 and a maximum learning rate of 3e-3.

The training data are sampled such that KID+, KID- and discarded day 2 embryos represent 20%, 10%, and 10% of a batch, respectively. The remaining 60% of the batch are sampled from KID+, KID- and discarded day 3 embryos that each represent 30%, 15%, and 15%, respectively. We use a batch size of 64 and focal loss [27] with an α of 0.5 and a γ of 2.0. In the loss function, KID+ are represented by values of 1, and KID- and discarded embryos are represented by values of 0. Each teacher model is trained with 7,800 batches. The student model is trained with 9,720 batches in the same manner as described for the day 5+ model, above. The results of the day 2/3 model are available in Table 2a.

Direct cleavage model

During development of the day 2/3 model, 5-fold cross-validation was used to evaluate its performance on different embryo subgroups. Here, it was found that direct cleavages were given unreasonably high scores, probably due to low occurrence in the transferred day 2 and 3 embryos, as these embryos are typically discarded. To test the hypothesis, we manually zeroed the output scores of the day 2/3 model for all DC13 and DC25 embryos. When doing so, similar AUCs were seen for both *All* and *KID* embryos between the day 2/3 model and KIDScore D3. Therefore, a separate model was developed to detect direct cleavages along with a combination model to balance the direct cleavage scores and the original day 2/3 scores.

The direct cleavage model is trained on manually annotated embryos from the training set in Table 1b to predict the presence of direct cleavages. A direct cleavage from 1 to 3 cells (DC13) is defined as $t_3 - t_{\text{PNf}} < 7.6$ hpi, where t_3 and t_{PNf} denote the timing of cell division to 3 cells and pronuclear fading, respectively. A direct cleavage from 2 to 5 cells (DC25) is defined as $t_5 - t_3 < 5.0$ hpi, where t_5 denotes the timing of cell division to 5 cells. The dataset for direct cleavages is described in Table S1.

	Day 2	Day 3	Day 5+	Total
No DC	722	7,939	46,413	55,074
DC13	452	1,248	6,599	8,299
DC25	615	1,016	5,326	6,957
Total	1,789	10,203	58,338	70,330

(a) Training data

	Day 2	Day 3	Day 5+	Total
No DC	210	1,766	11,526	13,502
DC13	133	260	1,579	1,972
DC25	163	232	1,357	1,752
Total	506	2,258	14,462	17,226

(b) Validation data

Table S1. Datasets used for developing the direct cleavage model and combination model. Both the (a) training data (80%) and (b) validation data (20%) are subsets of the original training dataset in Table 1a.

The architecture of the direct cleavage model is visualized in Figure S1. It consists of a MobileNetV2 [28] backbone applied to 1 frame/hour from 20–84 hp, with shared weights across all frames. The output for each frame ($8 \times 8 \times 1280$) is then spatially averaged ($1 \times 1 \times 1280$) and temporally concatenated into a 64×1280 feature vector. This feature vector is passed into two fully convolutional networks, one for predicting DC13 and one for predicting DC25. Each network consists of 7 one-dimensional convolutional layers with kernel sizes $[1,4,4,4,4,4,1]$ and output channels $[128,128,64,32,32,32,1]$. The last layer uses a sigmoid activation function, whereas all other layers use the rectified linear unit. Finally, the maximum value along the temporal output vector (49×1) represents the prediction score of either DC13 or DC25.

The DC model is trained using the Adam optimizer [25] with β_1 and β_2 set to 0.9 and 0.999, respectively, with a one-cycle learning rate schedule [26] and an initial learning rate of 1e-4 and a maximum learning rate of 1e-3. We sample random frame sequences of 16 frames from all videos in the training dataset and label them as DC13 and/or DC25 if the respective direct cleavage is fully visible in the frames according to the embryologist annotations. The training data are sampled such that non direct cleavages, DC13 and DC25 represent 50%, 25% and 25% of a batch, respectively. We use a batch size of 24, 15% dropout, and a loss function consisting of the sum of two binary cross-entropy losses, one for each output. The model is trained with 52,200 batches.

Table 1b describes the test set used to evaluate the direct cleavage model as well as two confusion matrices, showing classification performance for DC13 and DC25, individually. The model achieves an accuracy of 92% and a AUC of 0.95 for DC13, and an accuracy of 90% and a AUC of 0.88 for DC25.

	Day 2	Day 3	Day 5+	Total
No DC	146	1,718	10,103	11,967
DC13	111	215	1,488	1,814
DC25	142	223	1,177	1,542
Total	399	2,156	12,768	15,323

(a) Test data

Annotation \ Prediction	No DC	DC13
No DC	12,913	442
DC13	753	1,215

(b) DC13 confusion matrix

Annotation \ Prediction	No DC	DC25
No DC	13,403	224
DC25	1,277	419

(c) DC25 confusion matrix

Table S2. Test data and test results for the direct cleavage model. The test data in (a) is a subset of the original test dataset in Table 1b. (b) and (c) show confusion matrices of thresholded model predictions for the DC13 and DC25 outputs, individually. Thresholds are chosen to maximize accuracy on the validation data in Table S1b.

Combination model

To combine the scores of the day 2/3 model and the direct cleavage model, a multivariate logistic regression model is developed. Due to the rare presence of direct cleavages in the training set of day 2 and 3 KID embryos, we use day 5+ KID embryos from

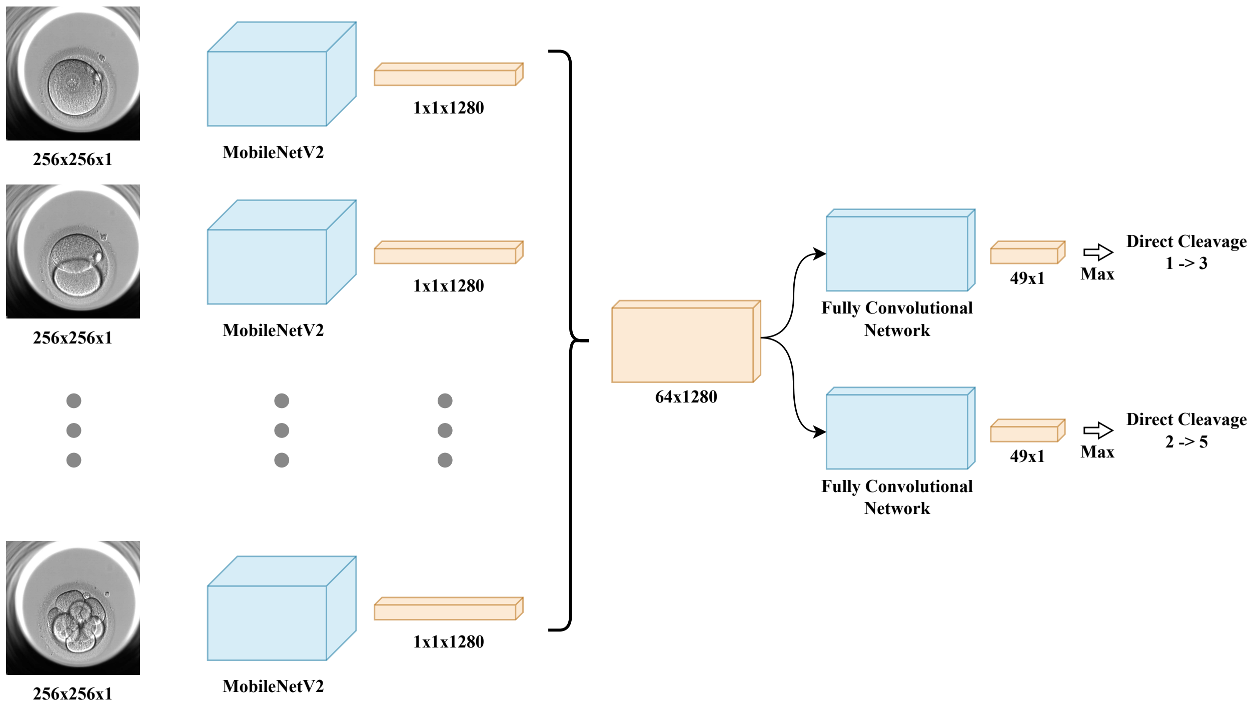

Figure S1. Architecture overview of model that predicts whether there is a direct cleavage from one to three cells or from two to five cells.

the training set in Table 1a to estimate the parameters of the logistic model. This is because transferred embryos on day 5+ in general include more direct cleavages than on day 2 or 3, since blastocyst presence outweighs cleavage stage morphokinetic parameters such as DC13 and DC25 in most selection strategies.

For estimation of the logistic parameters, the KID outcome is the independent variable, whereas model predictions are predictors that are first extracted for both the day 2/3 model ($y_{\text{Day 2/3}}$) and the direct cleavage model (y_{DC13} and y_{DC25}). For calculating day 2/3 model scores, day 5+ image sequences are truncated by multiples of 24 hours to resemble day 2 or day 3 image sequences. multiples of 24 hours are subtracted from the day 5+ image sequences to resemble day 2 and 3 sequences. The resulting model is given by:

$$p\left(y_{\text{Day 2/3}}, y_{\text{DC13}}, y_{\text{DC25}}\right) = \frac{1}{1+\exp\left(-4.86+5.28y_{\text{Day 2/3}} - 1.28y_{\text{DC13}} - 1.15y_{\text{DC25}}\right)} \tag{2}$$

The results of the combination model are available in Table 2a and Table 2b.

A.3 Calibration

In order to facilitate the use of iDAScore v2.0 for both ranking embryos within a cohort and predicting chances of pregnancy, we calibrate the score output to better match implantation rates [1]. We calibrate the model separately for each day (2, 3, and 5+) on single embryo transfers on the training set in Table 1a. The models are calibrated using Platt scaling [29] which is based on a logistic regression model. The calibration curves are shown in Figure 3.

Since implantation probabilities depend not only on embryo characteristics but also patient demographics and clinical practice, calibration performance may not be generalizable across different subgroups. Therefore, to avoid confusion between patient-wide and patient-specific probabilities, the calibrated scores are ultimately linearly scaled to the range 1.0–9.9, which is the final output of iDAScore v2.0.

Supplementary references

23. Carreira, J. & Zisserman, A. Quo vadis, action recognition? a new model and the kinetics dataset. In *2017 IEEE Conference on Computer Vision and Pattern Recognition (CVPR)*, 4724–4733 (2017).

24. Hinton, G., Vinyals, O. & Dean, J. Distilling the knowledge in a neural network (2015). https://arxiv.org/abs/1503.02531.

25. Kingma, D. P. & Ba, J. Adam: A method for stochastic optimization (2014). https://arxiv.org/abs/1412.6980.

26. Smith, L. N. Cyclical learning rates for training neural networks. In *2017 IEEE Winter Conference on Applications of Computer Vision (WACV)*, 464–472 (2017).

27. Lin, T.-Y., Goyal, P., Girshick, R., He, K. & Dollár, P. Focal loss for dense object detection. In *2017 IEEE International Conference on Computer Vision (ICCV)*, 2999–3007 (2017).

28. Sandler, M., Howard, A., Zhu, M., Zhmoginov, A. & Chen, L.-C. Mobilenetv2: Inverted residuals and linear bottlenecks. In *2018 IEEE/CVF Conference on Computer Vision and Pattern Recognition*, 4510–4520 (2018).

29. Platt, J. Probabilistic outputs for support vector machines and comparisons to regularized likelihood methods. *Adv. Large Margin Classif.* **10** (2000).

B Data description

Table S3. Distribution by clinic of day 2 embryos.

Clinic	Mean age	ICSI	IVF	SingleET	MultiET	Fresh	Thawed	Discarded	KID-	KID+
1	36.0	675	0	48	148	196	0	479	180	16
10	37.3	525	55	120	47	99	68	413	143	24
11	39.4	62	10	10	12	22	0	50	20	2
16	37.8	2,324	1,709	457	873	1,330	0	2,703	1,193	137
17	32.4	2,038	2,633	458	394	852	0	3,819	744	108
18	37.9	4,249	3,551	769	1,456	2,217	8	5,575	2,035	190
19	33.5	803	264	199	168	367	0	700	290	77
20	36.7	4,311	6	3,868	0	3,440	428	449	2,957	911
22	-	893	519	944	0	944	0	468	698	246
Total	36.9	15,880	8,747	6,873	3,098	9,467	504	14,656	8,260	1,711

Table S4. Distribution by clinic of day 3 embryos.

Clinic	Mean age	ICSI	IVF	SingleET	MultiET	Fresh	Thawed	Discarded	KID-	KID+
1	36.1	3,994	8	254	725	979	0	3,023	834	145
4	35.5	83	53	28	48	76	0	60	66	10
8	38.6	159	51	58	50	88	20	102	93	15
10	37.5	170	31	28	16	38	6	157	36	8
11	38.5	127	14	26	16	31	11	99	38	4
16	36.4	1,429	1,357	343	534	877	0	1,909	754	123
17	33.1	1,910	2,125	1,143	688	1,831	0	2,204	1,430	401
18	36.6	4,300	4,320	808	1,372	2,173	7	6,440	1,832	348
19	32.9	2,403	886	430	276	705	1	2,583	516	190
20	37.9	145	1	111	0	98	13	35	86	25
Total	35.2	14,720	8,846	3,229	3,725	6,896	58	16,612	5,685	1,269

Table S5. Distribution by clinic of day 5+ embryos.

Clinic	Mean age	ICSI	IVF	SingleET	MultiET	Fresh	Thawed	Discarded	KID-	KID+
1	34.1	10,593	34	422	655	1,077	0	9,550	777	300
2	35.5	737	14	65	12	77	0	674	46	31
3	35.4	7,265	210	403	63	324	142	7,009	309	157
4	33.9	665	634	162	54	167	49	1,083	131	85
5	37.4	4,008	2,214	759	144	418	485	5,319	634	269
6	34.5	987	1,216	136	48	81	103	2,019	150	34
7	35.8	1,829	1,609	513	45	345	213	2,880	396	162
8	36.1	1,491	967	542	15	387	170	1,901	389	168
9	38.1	231	342	90	16	67	39	467	67	39
10	37.3	11,741	4,995	1,982	407	1,065	1,324	14,347	1,677	712
11	36.9	5,791	828	702	263	689	276	5,654	747	218
12	35.3	0	386	32	39	71	0	315	54	17
13	36.7	13	2,894	505	322	429	398	2,080	588	239
14	36.9	68	621	148	18	100	66	523	115	51
15	37.1	642	317	181	9	76	114	769	141	49
16	36.3	4,180	4,742	515	304	819	0	8,103	583	236
17	32.5	7,827	12,723	1,043	66	1,090	19	19,441	649	460
18	36.6	6,434	5,936	1,236	183	550	869	10,951	940	479
19	33.2	5,864	5,433	621	89	542	168	10,587	407	303
20	40.0	3,879	10	2,535	2	42	2,495	1,352	1,847	690
21	36.0	12,828	37	993	423	47	1,369	11,449	630	786
Total	35.8	87,073	46,162	13,585	3,177	8,463	8,299	116,473	11,277	5,485

Supplementary Results

C Subgroup analysis

Table S6. Subgroup AUCs for KID embryos in test set transferred on day 2, 3 and 5+. Subgroups with less than 100 KID embryos were not evaluated.

		Day 2		Day 3		Day 5+	
Parameter	Subgroup	N	AUC	N	AUC	N	AUC
Age	< 30	100	.650 [.535-.765]	151	.584 [.490-.678]	235	.597 [.525-.669]
	30-34	315	.706 [.644-.767]	284	.594 [.521-.666]	564	.685 [.642-.729]
	35-39	447	.640 [.577-.702]	313	.598 [.514-.681]	687	.655 [.613-.697]
	> 39	425	.723 [.627-.819]	248	.743 [.648-.838]	684	.772 [.732-.812]
Insemination method	ICSI	998	.654 [.615-.694]	599	.630 [.574-.686]	1723	.719 [.694-.743]
	IVF	425	.662 [.588-.736]	404	.609 [.547-.671]	699	.678 [.637-.718]
Transfer protocol	Cryopreserved	64	-	8	-	1223	.685 [.655-.715]
	Fresh	1359	.674 [.639-.709]	995	.623 [.582-.665]	1199	.731 [.702-.760]
Year	< 2015	427	.626 [.543-.710]	409	.649 [.588-.710]	116	.604 [.493-.715]
	2015-2016	177	.730 [.650-.810]	286	.644 [.564-.725]	267	.708 [.645-.770]
	2017-2018	591	.642 [.591-.693]	250	.562 [.469-.654]	1360	.695 [.667-.724]
	> 2018	228	.552 [.469-.634]	58	-	679	.749 [.712-.786]
Clinic	1	27	-	141	.558 [.454-.662]	157	.781 [.698-.863]
	5	-	-	-	-	115	.734 [.641-.827]
	10	26	-	4	-	336	.685 [.626-.745]
	11	5	-	7	-	153	.731 [.643-.818]
	13	-	-	-	-	115	.651 [.547-.754]
	16	158	.703 [.563-.842]	106	.629 [.503-.756]	109	.753 [.654-.853]
	17	145	.601 [.472-.730]	248	.657 [.578-.736]	177	.670 [.591-.749]
	18	310	.641 [.535-.747]	334	.610 [.536-.684]	169	.593 [.504-.683]
	19	53	-	129	.640 [.512-.769]	105	.719 [.620-.818]
	20	568	.596 [.545-.647]	19	-	412	.781 [.734-.828]
	21	-	-	-	-	218	.517 [.440-.594]
	22	131	.526 [.417-.636]	-	-	-	-
